# Development and validation of five behavioral indices of flood adaptation

**DOI:** 10.1186/s12889-019-6564-0

**Published:** 2019-02-28

**Authors:** Pierre Valois, Maxime Caron, Anne-Sophie Gousse-Lessard, Denis Talbot, Jean-Sébastien Renaud

**Affiliations:** 1 Faculty of Education, Université Laval, 2320, rue des Bibliothèques, Quebec City, QC G1V 0A6 Canada; 2 Faculty of Medicine, Université Laval, 1050 Avenue de la Médecine, Quebec City, QC G1V 0A6 Canada

**Keywords:** Adaptation, Climate change, Index, Validation, Flooding

## Abstract

**Background:**

In the current context of climate change, climate forecasts for the province of Quebec (Canada) are a lengthening of the thunderstorm season and an increase in episodes of intense precipitations. These changes in the distribution of precipitations could heighten the intensity or frequency of floods, a natural hazard that concerns 80% of Quebec’s riverside municipalities. For the health and safety of the at-risk population, it is very important to make sure they have acquired necessary adaptive behaviors against flooding hazard. However, there has been no assessment of these flood adaptation behaviors to date. Thus, the aim of this study was to develop and validate five indices of adaptation to flooding.

**Methods:**

A sample of 1951 adults completed a questionnaire by phone. The questionnaire, specifically developed for this study, measured whether they did or did not adopt the behaviors that are proposed by public health officials to protect themselves against flooding.

**Results:**

The results of the item, confirmatory factor, and multiple correspondence analyses contributed to the development of five indices corresponding to the adaptation behaviors to adopt according to the chronology of events: (a) pre-alert preventive behaviors, (b) behaviors to carry out after the alert is issued, (c) behaviors to adopt during a flood not requiring evacuation, (d) behaviors to adopt during a flood requiring evacuation, and (e) post-flood behaviors. The results of this study also showed that people who perceive a risk of flooding in their home in the next 5 years tend to adopt more preventive behaviors and adaptation behaviors than those who perceive little or no risk at all. They also reveal that people who feel more adverse effects on their physical or mental health tend to adopt more adaptive behaviors than those who feel little or no adverse effects on their health.

**Conclusion:**

Across a series of psychometric analyses, the results showed that these flood adaptation indices could properly measure a vast range of adaptive behaviors according to the chronology of events. Therefore, researchers, public health agencies, and professionals can use them to monitor the evolution of individuals’ adaptive behaviors during floods.

**Electronic supplementary material:**

The online version of this article (10.1186/s12889-019-6564-0) contains supplementary material, which is available to authorized users.

## Background

There are many health problems related to flood situations. On the one hand, there can be physical problems, such as wounds, trauma, drowning, mold-induced respiratory problems, gastrointestinal diseases, leptospirosis, skin infections caused by contaminated waters, carbon monoxide poisoning, and electrocutions [1–9]. On the other hand, research has shown that there are also mental health problems, such as post-traumatic stress disorder (PTSD), anxiety, and depression, associated with flooding [2, 10–12]. For example, in 2011, in Brisbane, Australia, flood victims reported, among other things, respiratory problems, poor quality of sleep, psychological distress, and PTSD symptoms [13]. After a heavy rainfall in Southern Alberta (province in Western Canada), which affected approximately 100,000 people, a significant increase in injuries and in the average weekly administration of tetanus post-exposure prophylaxis was detected. In Saguenay (Quebec), floods that occurred in 1996 resulted in the death of 10 people and required the evacuation of 15,825 others [14]. In 2011, spring floods in the province of Quebec affected 40 municipalities. Among them, 11 declared a state of emergency, 2524 primary residences were flooded, 3927 persons were affected, 1651 were evacuated, and 7000 psychosocial interventions took place [15].

In this context, adaptation to climate change, particularly to flooding, is fundamental. Behavioral adaptation of individuals living in flood-prone areas is defined as behavioral adjustments intended to lessen the adverse health effects and the potential property damages. The need to establish structures that foster better adaptation by the population is increasingly acknowledged at the global scale [16]. Indeed, effective preparations reduce households’ vulnerability, decrease the impacts, shorten the post-disaster recovery period, and improve community resilience [17].

Municipal officials and public health authorities try to raise the population’s awareness of these issues by disseminating information on various protection measures for at-risk citizens. Those measures may concern the level of disaster preparedness, modifying the homes to make them more water-resistant, as well as the adoption of safe behaviors during and after a flood. For public health monitoring and promotion, it would thus be important to summarize these many diverse behaviors for coping with flooding. The creation of a composite index is thus warranted here [18]. The number of composite indices worldwide increases every year [19]. Their popularity stems from the fact that they illustrate issues that are complex and sometimes difficult to grasp (e.g., environment, poverty) while using a smaller set of indicators without losing the underlying basic information.

The aim of this study was thus to develop and validate five flood adaptation indices for people living in or bordering a flood zone. These indices correspond to the adaptation behaviors to adopt according to the chronology of events: (a) pre-alert preventive behaviors, (b) behaviors to carry out after the alert is issued, (c) behaviors to adopt during a flood not requiring evacuation, (d) behaviors to adopt during a flood requiring evacuation, and (e) post-flood behaviors. To our knowledge, no such indices of flood adaptation behaviors have been developed to date. Yet, Rufat, Tate, and Maroof [20] have previously raised the need to differentiate the phases of a flood disaster when creating indicators. These five indices are necessary because, in addition to targeting different adaptation behaviors, they are intended for slightly different groups of individuals, not all, for instance, having experienced alerts, home evacuations, or flooding.

At each of these adaptation phases, the behaviors identified as the best indicators will be grouped together in a contextualized composite index that will make it possible to monitor the evolution of individual adaptation to floods over time in Quebec, Canada. We will first describe the common methodology used to develop and validate the five adaptation indices. Next, to better illustrate our data analysis strategy, we will present all the results of the first index (i.e., pre-alert preventive behaviors). We will also present the results of the other four indices, but as concisely as possible for the readers’ benefit. Detailed results for the last indices will be available in the Supplemental Materials Section.

## Methods

### Sample and sampling procedure

To develop and test the validity of the flood adaptation indices, we used a stratified sample. This helped to preserve the geographical distribution of the flood-risk zones throughout the province of Quebec (Canada), which covers 1,667,441 km^2^ (643,802 mile^2^) and has a population of 8.3 million. Hence, the number of participants recruited in each of the 17 administrative regions in the province was proportional to the number of households in these regions. The target population consisted of all 136,505 households whose main residence was in or near a designated flood-prone area, as per the Centre d’expertise hydrique du Québec [Quebec Water Expertise Center].

A total of 1951 individuals (women, 44.54%; men, 55.46%) were surveyed by a polling firm: 1450 lived in an at-risk area (flood recurrence: every 20 to 100 years), and 501 lived less than 150 m from a designated flood-prone area. The sample size was estimated according to Cochran’s formula [21]: a 95% confidence level, a maximal variance for a five-point scale (i.e., 4), and a precision level of 0.12.

The interviews used a questionnaire developed for the study and lasted an average of 21 min 18 s, and the response rate was 21.82%. When no contact was made on the first attempt, the interviewers made a maximum of nine callbacks (for a total of 10 attempts) at various times before rejecting the telephone number. A list of addresses retrieved from “Adresses Québec” (a database of all the addresses in the province of Quebec) was sent to the polling firm. Its staff matched these addresses to phone numbers. To answer the survey, respondents had to be responsible for the household, either financially or in matters of familial care. They answered for the entire household, but individual demographic data were collected. All respondents were aged 18 years or older (M = 57.29, SD = 14.27; 18–29 [*n* = 35], 30–39 [*n* = 202], 40–49 [*n* = 281], 50–59 [*n* = 484], 60–69 [*n* = 506], 70–79 [*n* = 361], more than 80 [*n* = 121]) and could converse in French or in English. Men made up 44.54% of the sample, while women made up 55.46%. The first language of most of the participants (98.26%) was French. For 32.44% of the participants, the highest education level obtained was a university degree; 30.09% reported an annual net income of CAD$40,000 or less, 29.21% an income of CAD$40,001–CAD$80,000, and 26.71% an income of CAD$80,001 or more.

### Five flood adaptation indices

Initially, 55 adaptive behaviors were considered as potential components of the flood adaptation indices: 24 behaviors that are recommended for before the alert (e.g., raise the doorsills) or for which knowledge is required if the need arises (e.g., know how to cut off the electricity), 10 behaviors to adopt at the time of the alert (e.g., cut off the electricity if requested by the authorities), four behaviors to perform during a flood not requiring an evacuation (e.g., boil the water or use bottled water), seven behaviors to adopt during a flood requiring an evacuation (e.g., use the route indicated by the authorities to evacuate the neighborhood), and 10 behaviors to adopt after the flood (e.g., sterilize all kitchen items contaminated by the flood water). The behaviors in question are listed in Tables 1 to 5 below. The presence of filter questions allowed respondents to answer only the questions that concerned them (for instance, not all had experienced an alert or a flood). All these items were selected from a review of the literature on health [e.g. 22–25] and recommendations from public health agencies [26–29].

### Index of pre-alert preventive adaptation

For this index, the target population was the entire group of respondents (*n* = 1951). The participants were asked to indicate, using a dichotomous Yes-No response format, whether they did or did not carry out each of the 24 behaviors proposed (see Table 1). Answers of respondents who did not have access to the water shut-off valve or the electrical panel (behaviors 6 and 7) were considered in the non-preventive category because it was impossible for these respondents to adopt these behaviors. For behavior 24, answers indicating that the respondent did not have access to a basement were grouped together under the non-preventive category because it went without saying that no valuable items could be stored in the basement.

**Table 1:** List of pre-flood preventive behaviors

1. Have a list of emergency telephone numbers	
2. Have an emergency kit	
3. Make a list of your belongings that could be used for a claim in case of flooding	
4. Make a plan for evacuating your home in case of emergency	
5. Make a plan for evacuating your neighborhood in case of emergency	
6. Know how to shut off the water	
7. Know how to cut off the electricity	
8. Inquire about how to better prepare for a flood or to make your home more flood-resistant	
9. Inquire about the consequences that a flood could have on your physical or mental health	
10. Waterproof the foundations	
11. Raise the door sills	
12. Raise the foundations	
13. Raise the baseboard heaters or electrical outlets on the walls	
14. Replace water-sensitive flooring	
15. Install a backwater valve	
16. Relocate the home elsewhere on the property	
17. Make other changes to the building (e.g., to the windows, the insulation, the walls, the ceiling; seal the cracks).	
18. Reduce the area of surfaces that are not waterproof (e.g., replace asphalt with stones or another finish that lets the water through)	
19. Change the landscape to help water runoff	
20. Do drainage work around the home	
21. Check to be sure the foundation drain is not blocked	
22. Make other changes to the property to make it more flood-resistant (e.g., plant trees and shrubs, put stones on the property or near the stream, make a dam or a barrier, develop the riverbank, relocate structures on the property)	
23. Own a water pump	
24. Store valuable items somewhere besides the basement	

### Index of adaptation at the time of the alert

The target population for this preventive index was the one having experienced a flood alert, that is, 627 of the 1951 respondents. For this index (at the time of the alert), participants were asked to indicate whether they did or did not perform each of the 10 behaviors proposed (see Table 2). They had to use a Yes-No response format to indicate if they did or did not adopt the adaptive behaviors. One item (behavior 3) asked respondents if they had blocked the basement drain. Answers indicating that respondents had a backwater valve were grouped under the adaptative modality. Finally, answers indicating that the people had no yard furniture, patio, or vehicle (behavior 1) were classified under the adaptive modality because these items could be considered as not having been exposed to the water.

**Table 2:** List of behaviors to perform at the time of a flood alert

1. Move your lawn or patio furniture or your vehicle to higher ground	
2. Store items or furniture higher or on a higher floor	
3. Block the basement drain	
4. Cut off the electricity if requested by the authorities	
5. Waterproof the doors and windows with plastic tape	
6. Block the outside air inlets like the one for the clothes dryer, the range hood, the air exchanger, etc.	
7. Put sandbags on the property	
8. Implement other measures to prevent the water from entering (e.g., board up the windows, prepare the water pump, etc.)	
9. Check regularly if the risk of flooding has increased or decreased	
10. Help your neighbors implement their protective measures like putting sandbags	

### Index of adaptation during a flood not requiring an evacuation

The sample used to test the metric qualities of this index consisted in 669 respondents who had been flooded but not evacuated. For two of the four behaviors to adopt during a flood (“boil the water or use bottled water” and “install a pump to evacuate the water from the home”; see Table 3), respondents had to choose between the following answers: (1) yes, I adopted this behavior and (2) no, I did not adopt this behavior. For the two other behaviors (wear rubber gloves to handle items in contact with the flood water; wear rubber boots to walk in the flood water), respondents indicated how frequently they adopted each adaptive behavior using a four-point ordinal scale ranging from (1) “I never did this” to (4) “I always did this.” The answers were then grouped into two categories: behaviors that people adopt (I usually did this; I always did this) and behaviors that people do not adopt (I sometimes did this; I never did this). We merged these response options to give the same weighting to each of the behaviors. Furthermore, additional analyses showed that considering these merges did not change our results.

**Table 3:** List of behaviors to carry out during a flood not requiring an evacuation

1. Boil the water or use bottled water	
2. Wear rubber gloves to handle items in contact with the flood water	
3. Wear rubber boots to walk in the flood water	
4. Install a pump to drain the water from the home	

### Index of adaptation during a flood requiring an evacuation

This index was tested with respondents who had previously had to evacuate their home due to a flood, that is, 126 of the 1951 respondents. For all behaviors to adopt during an evacuation (see Table 4), respondents had to choose between the following answers: (1) yes, I adopted this behavior and (2) no, I did not adopt this behavior. Regarding “registering with a temporary shelter if available,” participants could also indicate that no temporary shelter was available. If so, their answers were considered in the non-adaptive category because registering with a temporary shelter was not possible for them. For the behavior “using the route indicated by the authorities to evacuate the neighborhood,” the answers indicating that the person was not aware of this information were grouped under the non-adaptive modality. Finally, for the item asking respondents if they had brought their emergency kit when evacuating their home (behavior 1), the answers indicating that they did not have an emergency kit were assigned to the non-adaptive modality.

**Table 4:** List of behaviors to carry out when evacuating one’s home

1. Bring your emergency kit, including your medication	
2. Lock the doors	
3. Register with a temporary shelter if available	
4. Use the route indicated by the authorities to evacuate the neighborhood	
5. Tell your loved ones where you can be easily reached	
6. Put your pets in a secure location	
7. Wait for the authorities’ permission before returning home	

### Post-flood adaptation index

For the development of the post-flood index, 10 adaptive behaviors were considered (see Table 5). Only respondents who had their home flooded in the past were targeted for this index.. Among all the respondents, 432 had experienced this situation. They thus formed the sample used to validate our post-flood index.

**Table 5:** List of post-flood behaviors

1. Have the condition of the electrical installation and heating appliances checked	
2. Replace the refrigerator insulation if it is wet or replace the appliance	
3. Disinfect the contaminated rooms	
4. Sterilize all kitchen items contaminated by the flood water	
5. Discard items in contact with the flood water	
6. Wear rubber gloves to handle items in contact with the flood water	
7. Check if mold has developed	
8. Make a list of the damages caused to the home and to your belongings	
9. Update your emergency kit	
10. Attend citizens’ meetings concerning the flood	

For six of the 10 behaviors to adopt after the flood (behaviors 1, 3, 4, 7, 8, 9; see Table 5), the respondents had to choose between the following answers: (1) yes, I did adopt this behavior and (2) no, I did not adopt this behavior.

For two questions, the answers were coded on a scale with three choices of answers: (a) “Did you attend citizens’ meetings concerning the flood” (behavior 10), for which the answer options were (0) attended no citizens’ meetings, (1) attended one citizens’ meeting, (2) attended two or more citizens’ meetings; and (b) “Did you replace the refrigerator insulation or change the appliance if it was wet” (behavior 2), for which the answer options were (0) No, I did not replace the insulation or change the appliance, but it did get wet, (1) It did not get wet, so I didn’t have to change the insulation or the appliance, (2) Yes, I changed the insulation or the appliance because it got wet. The answers were then grouped into two categories: behaviors that people adopt (response options 1 and 2) and behaviors that people do not adopt (response option 0).

Finally, for the two last behaviors, the answers were coded on a scale with four choices of answers: (a) “discard items in contact with the flood water” (behavior 5), for which the answer options were (0) I discarded no items, (1) a few, (2) most of them, (3) all the items; and (b) “wear rubber gloves to handle items in contact with the flood water” (behavior 6), for which the answer options were (0) never, (1) sometimes, (2) most of the time, (3) always. The answers were then grouped into two categories: behaviors that people adopt (response options 2 and 3) and behaviors that people do not adopt (response options 0 and 1).

### Variables theoretically related to flood adaptation indices

Two variables were also measured and correlated with the indices to test their validity. In this study, these variables corresponded to the adverse health impacts felt during or after a flood and the perceived risk of being flooded in the next 5 years.

### Self-reported adverse health impacts

The answers to two questions were combined to create this variable: “Was your physical health adversely affected by the flood?” and “Was your mental health adversely affected by the flood?” Those who reported feeling “a moderate number of” or “many” adverse effects for at least one of these two questions were defined as the group feeling the most at risk. Those who reported feeling “no” or “a few” adverse effects to the two questions were defined as the group feeling the least at risk.

### Self-reported perceived risk

The second variable used was the perceived risk of being flooded in the coming years: “In your opinion, what is the risk of your current home being flooded in the next five years?” This question was rated on a five-point ordinal scale: “very high,” “high,” “moderate,” “low,” “very low,” or “nil.” People who reported that the risk was “very high,” “high,” or “moderate” formed the group feeling the most at risk of their current home being flooded in the next 5 years. Those who reported that the risk was “low,” “very low,” or “nil” formed the group feeling the least at risk.

### Statistical analyses

Before performing statistical analyses, we reweighted the data to compensate for imbalances between the proportions of respondents in each administrative region in the sample and those of the target population [30]. Because there were some missing values for age and education level, we imputed the missing data for these variables employing predictive mean matching [31]. Then, for each of the five indices, we conducted four series of statistical analyses.

First, to assess the psychometric qualities of the adaptation indices, we performed an item analysis*,* using Samejima’s graded response model [32]. The objective of this analysis was to assess the performance of the items according to a certain number of psychometric parameters (e.g., the power to differentiate between individuals who adapt well to flooding and those who adapt less well) and to determine which items to retain in a measurement instrument, such as an index. In other words, the discriminant parameter could be conceived as a description of the association between the item and the measured construct. This is because the higher the discrimination index for an adaptation measuring item, the more that item is able to discriminate between individuals who adapt well to flooding and those who adapt less well. We used Baker’s [33] guidelines to interpret discrimination power: (a) very poor: 0.34 or less, (b) poor: 0.35–0.64, (c) moderate: 0.65–1.34, (d) good: 1.35–1.69, (e) very good: 1.70 or higher.

Second, we conducted a confirmatory factor analysis to assess the unidimensionality of the flood adaptation index. We tested a model that included all the behaviors within a single construct representing flood adaptation. We then assessed the compatibility of the empirical data with the hypothetical measurement model. To do so, we used various fit indices, including the comparative fit index (CFI), the Tucker–Lewis index (TLI), the ratio of the chi square to its degrees of freedom (χ^2^/*df*), and the root mean square error of approximation (RMSEA). Researchers generally agree that the model fit is acceptable or excellent if the CFI and the TLI exceed 0.90 or 0.95 with the χ^2^/*df* below 5 or 2 and the RMSEA below 0.08 or 0.06, respectively [34, 35].

Third, we conducted a multiple correspondence analysis, a data reduction procedure [36] that is frequently performed in the construction of composite indices [18, 37]. The percentage of inertia computed by applying Greenacre’s [38] method was used to countervalidate the result of the confirmatory factor analysis regarding the unidimensionality of the flood adaptation index. The multiple correspondence analysis results were also interpreted in terms of the contribution of the active variables (here, the measured behaviors) to the factorial dimensions obtained through the multiple correspondence analysis.

Finally, once the psychometric properties of an adaptation index were confirmed, we conducted a validity analysis. The essential function of a validity analysis is to determine the relationship between test results (here, the scores on each flood adaptation index) and a variable theoretically related to the construct studied [18, 39]. In this study, two criterion variables were used: self-reported adverse health impacts and self-reported perceived vulnerability. The choice of these variables depended on the target population. When the index to be created concerned only the population having experienced a flood, the variable used corresponded to the adverse health impacts felt during a flood. In the case where the index developed targeted the people whose home had been flooded, those living in a flood-prone zone but whose home had never been flooded, and those living on the border of a flood-prone zone, the variable used was the perceived risk of being flooded in the coming years.

To prove concept validity, we used two statistical methods. First, each index was correlated (tetrachoric correlation) with its respective related variables (perceived adverse health impacts or perceived vulnerability). Second, the prevalence of each associated variable was compared in terms of adaptation levels using the odds ratio.

## Results

As previously mentioned, we will describe in detail the reliability and validity results of the first index (i.e., pre-alert preventive behaviors), followed by a more succinct presentation of the results concerning the four other indices to lighten the text. Detailed results for the last four indices will be available in the Supplemental Materials Section.

### Item analysis

#### Index of pre-alert preventive adaptation

***The results of the item analysis using*** the Excel add-in EIRT [40] revealed that this index had good reliability. In fact, the majority of the 24 adaptive behaviors appeared to properly measure the adoption of pre-alert preventive adaptation behaviors, that is, to discriminate between individuals who adapt well to flooding and those who adapt less well according to Baker’s [33] guidelines. Only one behavior, “store valuable items somewhere besides the basement,” seemed problematic, with a discrimination index approaching zero (0.003; see Table 6). This item was therefore removed from the index. The reason for this low discrimination power was not that almost all respondents reported adopting this behavior (i.e., a low response variability): 30.42% of respondents did not do so compared with 69.58% who did. The reason was rather that the participants believed it was possible to protect valuable items by storing them in a high spot in the basement.

**Table 6:** Discrimination indices for each preventive behavior

Adaptive behaviors	Discrimination index	99% CI	Mean
1. Have a list of emergency telephone numbers	1.162	[0.979–1.345]	0.224
2. Have an emergency kit	1.540	[1.385–1.695]	0.776
3. Make a list of your belongings that could be used for a claim in case of flooding	0.741	[0.606–0.876]	0.321
4. Make a plan for evacuating your home in case of emergency	0.624	[0.495–0.753]	0.415
5. Make a plan for evacuating your neighborhood in case of emergency	0.951	[0.778–1.123]	0.199
6. Know how to shut off the water	0.593	[0.429–0.757]	0.841
7. Know how to cut off the electricity	0.800	[0.566–1.035]	0.925
8. Inquire about how to better prepare for a flood or to make your home more flood-resistant	1.219	[1.035–1.403]	0.246
9. Inquire about the consequences that a flood could have on your physical or mental health	0.933	[0.744–1.121]	0.147
10. Waterproof the foundations	1.486	[1.289–1.684]	0.306
11. Raise the door sills	1.308	[1.105–1.511]	0.189
12. Raise the foundations	1.292	[1.078–1.506]	0.153
13. Raise the baseboard heaters or electrical outlets on the walls	1.368	[1.158–1.578]	0.187
14. Replace water-sensitive flooring	1.165	[0.978–1.352]	0.206
15. Install a backwater valve	0.901	[0.758–1.043]	0.511
16. Relocate the home elsewhere on the property	0.816	[0.370–1.262]	0.017
17. Other changes made to the building	0.716	[0.425–1.008]	0.041
18. Reduce the area of surfaces that are not waterproof	0.931	[0.719–1.144]	0.109
19. Change the landscape to help water runoff	0.949	[0.798–1.100]	0.349
20. Do drainage work around the home	1.069	[0.907–1.231]	0.319
21. Check to be sure the foundation drain is not blocked	1.034	[0.882–1.186]	0.443
22. Make other changes to the property to make it more flood-resistant	0.347	[0.077–0.618]	0.044
23. Own a water pump	0.770	[0.635–0.905]	0.536
24. Store valuable items somewhere besides the basement	0.003	[−0.118–0.123]	0.581

The results in Table 6 also show that the most adopted behaviors were: “know how to cut off the electricity” (92.49%) and “know how to shut off the water” (84.07%). Conversely, “relocate the home elsewhere on the property” was the least adopted behavior (1.76%) and “make other modifications to the building” was the second least adopted (4.14%).

##### Index of adaptation at the time of the alert

The results of the item analysis (data not shown; see Additional file 1) indicated that all adaptive behaviors at the time of the alert could discriminate between people who adapt and those who do not adapt. The most discriminating behaviors were “put sandbags on the property” (1.67; good discriminating power), “block outdoor air inlets” (1.64; good discriminating power), and “help your neighbors implement their protective measures” (1.56; good discriminating power). The least discriminating indicators were “block the basement drain” (0.52), “check regularly if the risk of flooding has increased or decreased” (0.62), and “other measures to prevent water from entering the home” (0.66).

The results also showed that “take measures other than those mentioned to prevent water from entering the home” was not a behavior frequently reported by the respondents (2.94%). Among the other least reported behaviors were “block the outside air inlets like the one for the clothes dryer” (5.67%) and “waterproof the doors and windows with plastic tape” (9.37%). The most frequently adopted behavior (56.38%) was “check regularly if the risk of flooding has increased or decreased,” followed by “move the lawn or patio furniture or the vehicle to higher ground” (49.42%), and “store items or furniture higher or on a higher floor” (47.76%).

### Index of adaptation at the time of the flood not requiring an evacuation

An item analysis was performed on the four behaviors to adopt during a flood not requiring evacuation. The results (data not shown; see Additional file 2) suggested that these four behaviors aptly measured the concept of adaptation in the context of a flood not requiring evacuation. The item analysis revealed that the most discriminating behavior was “wear rubber gloves to handle items in contact with the flood water” (2.299; excellent discriminating power). The second most discriminating behavior was “wear rubber boots to walk in the flood water” (1.270, moderate discriminating power).

The most frequently performed behavior was “wear rubber boots to walk in the flood water” (67.93%) and the least performed was “wear rubber gloves to handle items in contact with the flood water” (24.70%).

### Index of adaptation during a flood requiring evacuation

The item analysis of the seven behaviors to carry out during an evacuation was performed with a non-parametric model (kernel density estimator [41]) because the sample of respondents having had to evacuate their home was too small to use a parametric model (*n* = 124). Two of the seven adaptive behaviors were not retained in the item analysis because they applied only to a very small proportion of the respondents: “place your pets in a safe location” and “register with a temporary shelter, if such a center is available to you.” This decision is justified because several people did not have pets (54 respondents on 124) or did not have access to temporary shelters (40 on 124).

The non-parametric item analysis models did not produce any item discrimination index but did provide item characteristic curves (data not shown; see Additional file 3). The curves obtained indicated that all five behaviors that were left had acceptable discrimination power; in other words, their item characteristic curve had a relatively steep positive slope. Thus, the following five behaviors were retained for the creation of the index: (1) bring your emergency kit; (2) lock the doors when leaving; (3) tell your loved ones where you can be easily reached; (4) use the route indicated by the authorities to evacuate the neighborhood, and (5) wait for the authorities’ permission before returning home.

The results also revealed that the following two behaviors were adopted by the majority of the respondents who had been evacuated: “lock the doors when leaving” (90.78%) and “tell your loved ones where you can be easily reached after the evacuation” (89.59%). The least performed behaviors were “register with a temporary shelter if available” (23.66%), and “bring your emergency kit, including your medication” (32.88%).

### Post-flood adaptation index

The results of the item analysis (data not shown; see Additional file 4) revealed that the 10 behaviors seemed to measure the post-flood adaptation construct. The most discriminating were: “have the condition of the electrical installation and heating appliances checked” (2.134, excellent discriminating power), “disinfect the contaminated rooms” (2.079, excellent discrimination power), and “ discard items in contact with the flood water” (1.934, excellent discrimination power). Conversely, the least discriminating behaviors were: “check if mold has developed” (0.314), “update your emergency kit” (0.384), and “replace the refrigerator insulation if it is wet or replace the appliance” (0.996).

The results also showed that the most reported behaviors were “disinfect the contaminated rooms” (77.11%; see Fig. 5) and “make a list of the damages caused to the home and to your belongings” (53.74%). The least frequently adopted behaviors were “check if mold has developed” (15.49%) and “attend at least one citizens’ meeting concerning the flood” (26.00%).

### Confirmatory factor analysis

We then tested the factorial validity of each index by determining whether the different categories of flood adaptation behaviors corresponded to each construct: (a) pre-alert flood adaptation, (b) adaptation during the alert, (c) adaptation during a flood not requiring evacuation, (d) adaptation during a flood requiring evacuation, and (e) post-flood adaptation.

### Index of pre-alert preventive adaptation

**Fig. 1:**
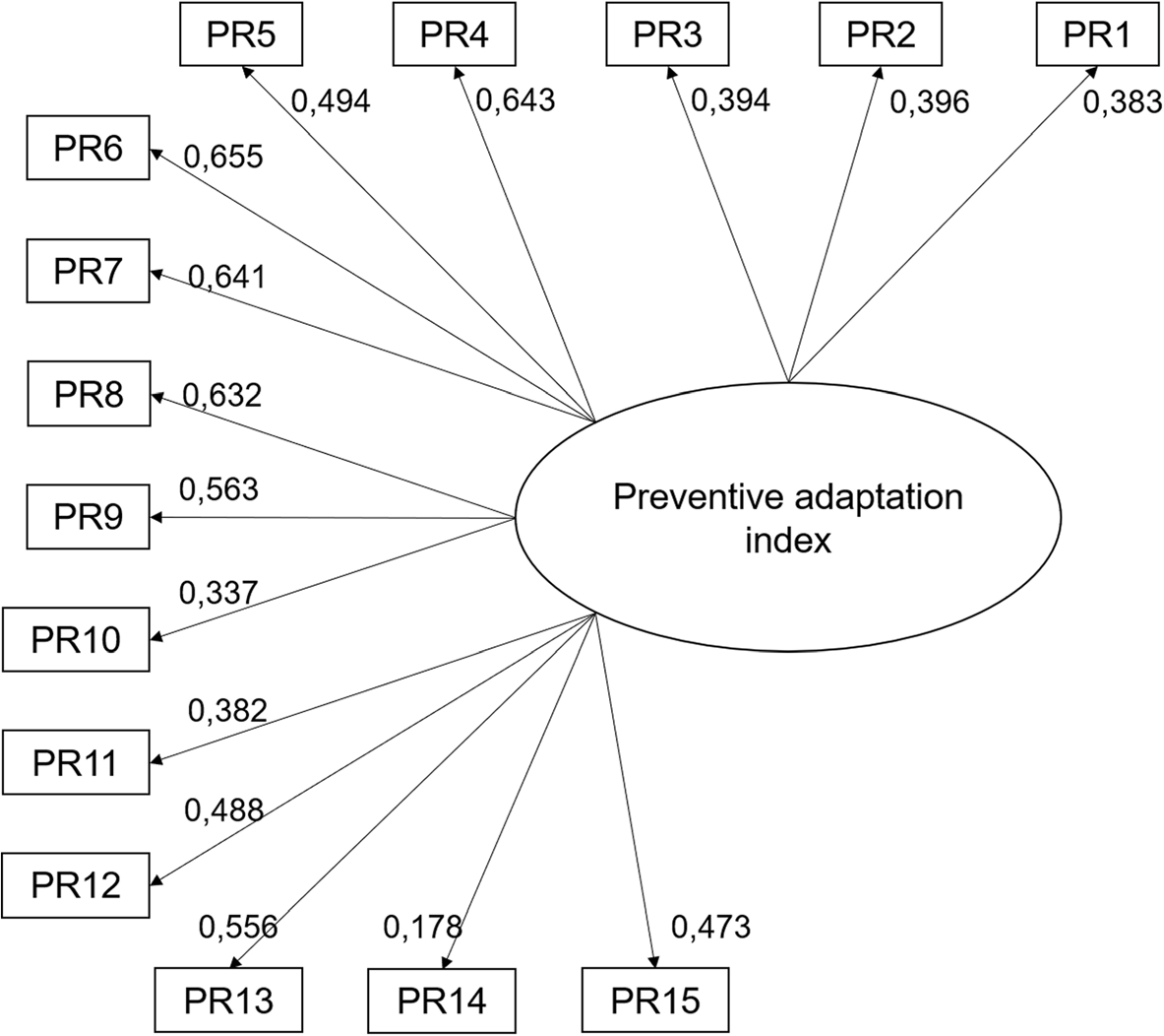
Final model for the preventive adaptation index tested by confirmatory factor analysis. Legend: PR1: Made a list of your belongings that could be used for a claim in case of flooding; PR2: Made a plan for evacuating your neighborhood in case of emergency; PR3: Know how to cut off the water or the electricity; PR4: Inquire about how to better prepare for a flood or to make your home more flood-resistant; PR5: Inquire about the consequences that a flood could have on your physical or mental health; PR6: Waterproof the foundations; PR7: Raise the baseboard heaters and electrical outlets on the walls; PR8: Replace water-sensitive flooring with a waterproof finish; PR9: Install a backwater valve; PR10: Relocate the home elsewhere on the property; PR11: Other changes made to the building; PR12: Change the landscape to help water runoff; PR13: Check to be sure the foundation drain is not blocked; PR14: Make other changes to the property to make it more flood-resistant; PR15: Own a water pump

The results showed a poor fit of the data with the theoretical model (CFI = 0.794, TLI = 0.773, RMSEA = 0.059) that included the 23 preventive behaviors to adopt before an alert. We therefore used the modification parameter estimates and indices provided in Mplus to identify potentially problematic indicators (i.e., loadings < 0.30; substantial correlate residual). The results showed that there were eight behaviors causing problems because they were too highly correlated with another behavior (data not shown; see Additional file 5). Consequently, these behaviors were deleted or paired with a correlated behavior. For instance, “make a plan for evacuating your home in case of emergency” was not retained in the index because its correlation with “make a plan for evacuating your neighborhood” was too high (r = 0.554), suggesting that these behaviors were redundant. The results showed that the revised model had an adequate level of fit to the data: CFI = 0.924, TLI = 0.911, χ^2^/*df =* 3.50, and RMSEA = 0.036. To further improve the index validity, we tested the revised model by combining these pairs of correlated behaviors. For example, we created a single variable that combined “know how to shut off the water” and “know how to cut off the electricity” to obtain a more general behavior called “know how to shut off the water or the electricity.” The results indicated that only this water-electricity combination improved the quality of the index. The final model therefore comprised 15 behaviors (behaviors 3, 5, 8, 9, 10, 13 to 17, 19, 21, 22, 23, and the combination of behaviors 6 and 7) and showed adequate fit to the data: CFI = 0.933, TLI = 0.921, RMSEA = 0.034 (see Fig. 1).

### Index of adaptation at the time of the alert

We also verified the factorial validity of the adaptation index comprising the 10 behaviors to carry out at the time of the alert. The results indicated that the values of two of the fit indices (CFI and TLI) were slightly below the desired minimal value (CFI = 0.893, TLI = 0.862 <  0.90). For its part, the RMSEA value indicated a good fit of the data with the model (RMSEA = 0.048).

**Fig. 2:**
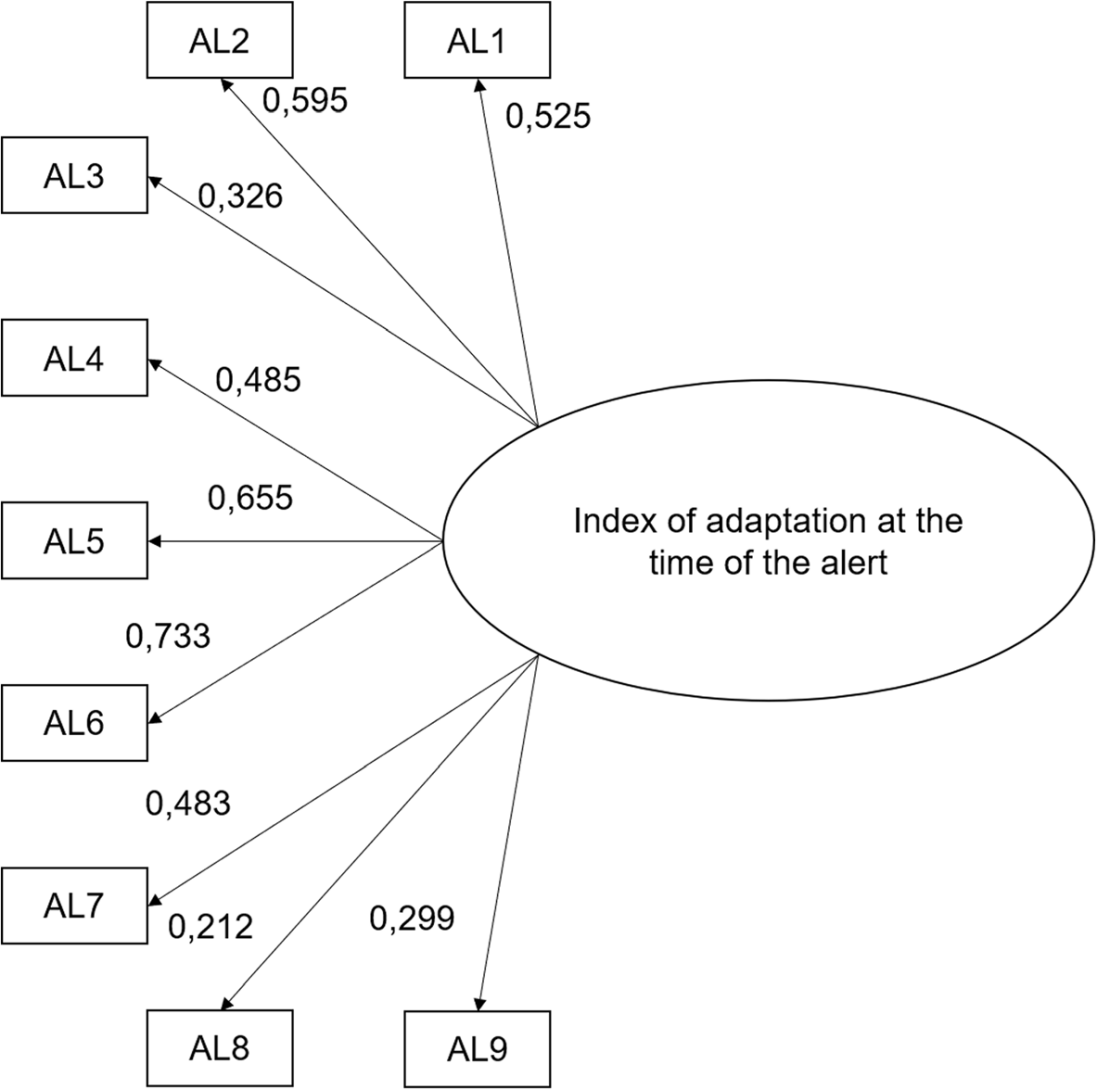
Model tested by confirmatory factor analysis for the index of adaptation at the time of the alert. Legend: AL1: Move your lawn or patio furniture or your vehicle to higher ground; AL2: Store items or furniture higher or on a higher floor; AL3: Block the basement drain; AL4: Cut off the electricity if requested by the authorities; AL5: Waterproof the doors and windows with plastic tape; AL6: Block the outside air inlets like the one for the clothes dryer, the range hood, the air exchanger, etc.; AL7: Put sandbags on the property or help your neighbors implement their protective measures; AL8: Implement other measures to prevent the water from entering; AL9: Check regularly if the risk of flooding has increased or decreased

The results showed that “help your neighbors implement their protective measures like putting sandbags” was too highly correlated with “put sandbags on the property” (r = 0.64). We decided to combine these two behaviors rather than eliminating one of them because the model fit was better: CFI = 0.932, TLI = 0.909, χ^2^/*df =* 1.71, RMSEA = 0.035 vs. CFI = 0.913, TLI = 0.884, χ^2^/*df =* 1.96, and RMSEA = 0.040). The final adaptation index thus contains nine items (see Fig. 2).

### Index of adaptation during a flood requiring evacuation

**Fig. 3:**
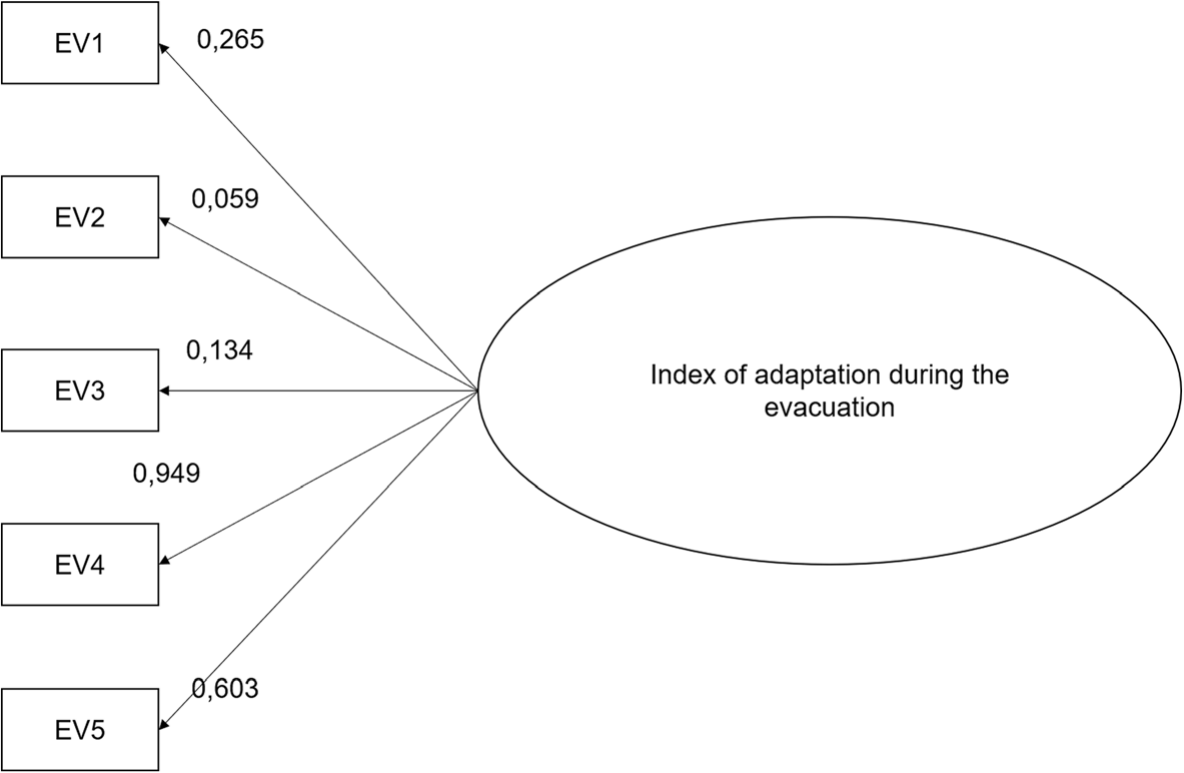
Final model tested by confirmatory factor analysis for the index of adaptation during an evacuation. Legend: EV1: Bring your emergency kit, including your medication; EV2: Lock the doors; EV3: Tell your loved ones where you can easily be reached; EV4: Use the route indicated by the authorities to evacuate the neighborhood; EV5: Wait for the authorities’ permission before returning home

A confirmatory factor analysis was then performed on the five behaviors to adopt during a flood requiring evacuation. The results indicated that the data fit well to the model (CFI = 1.000, TLI = 1.396, χ^2^/*df* = 0.39, and RMSEA = 0.000); see Fig. 3). However, the saturation coefficients associated with each of the behaviors were non-significant (*p* > 0.05). One potential explanation for this result is the small sample size used for the creation of this index because only 124 respondents had had to evacuate due to a flood.

### Index of adaptation during a flood not requiring evacuation

**Fig. 4:**
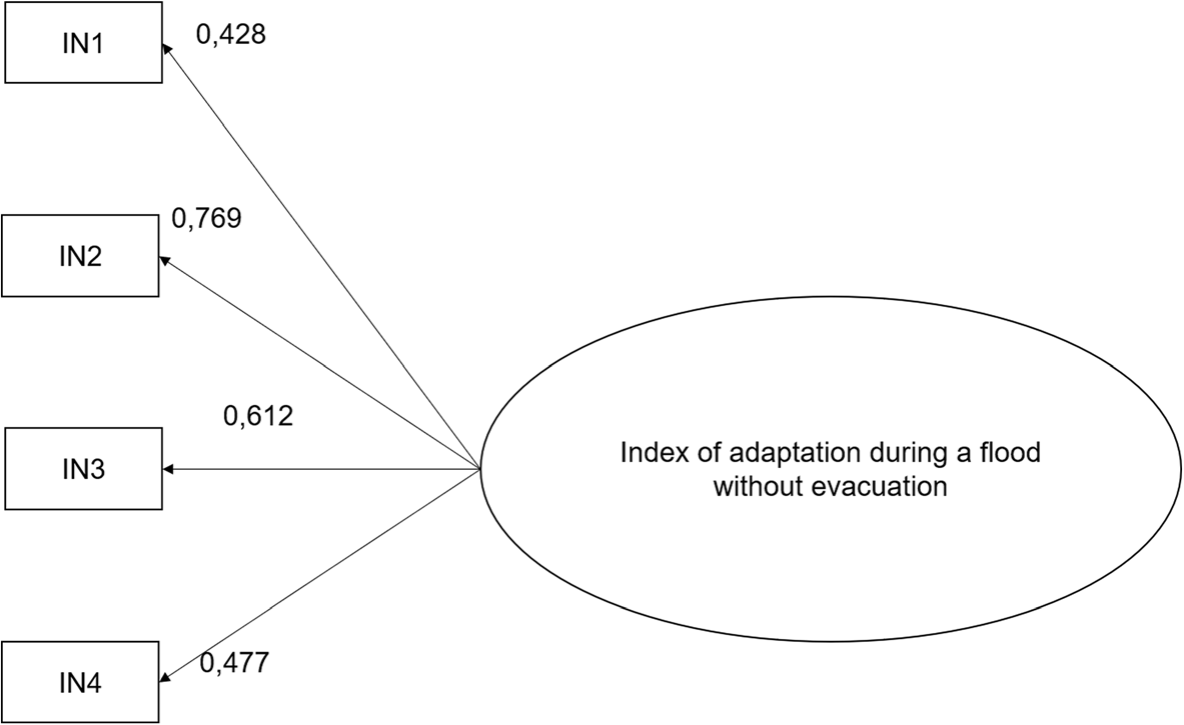
Final model tested by confirmatory factor analysis for the index of adaptation during a flood not requiring evacuation. Legend: IN1: Boil the water or use bottled water; IN2: Wear rubber gloves to handle items in contact with the water; IN3: Wear rubber boots to walk in flood water; IN4: Install a pump to drain the water from the home

Further to the item analysis, we conducted a confirmatory factor analysis to verify whether the adaptation behaviors to adopt at the time of a flood not requiring evacuation concerned one same construct. The results obtained showed that the model fit well with the data (CFI = 0.976, TLI = 0.928, χ^2^/*df* = 2.59, and RMSEA = 0.052; see Fig. 4) and that the saturation coefficients were all significant (*p* <  0.05).

### Post-flood adaptation index

**Fig. 5:**
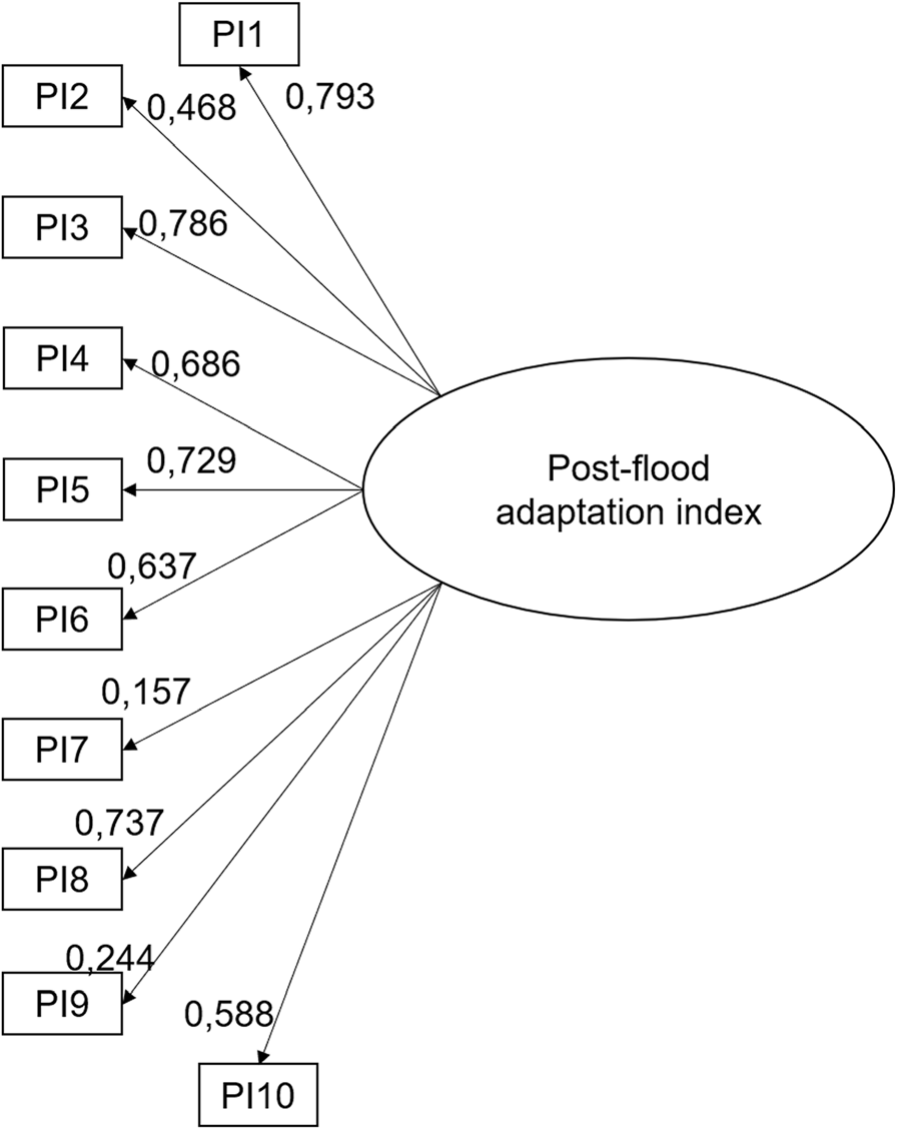
Final model tested by confirmatory factor analysis for the index of post-flood adaptation. Legend: PI1: Have the condition of the electrical installation and heating appliances checked; PI2: Replace the refrigerator insulation if it is wet or replace the appliance; PI3: Disinfect the contaminated rooms; PI4: Sterilize all kitchen items contaminated by the flood water; PI5: Discard items in contact with the flood water; PI6: Wear rubber gloves to handle items in contact with the water; PI7: Check if mold has developed; PI8: Make a list of the damages caused to the home and to your belongings; PI9: Update your emergency kit; PI10: Attend citizens’ meetings concerning the flood

The results of the confirmatory factor analysis of the 10 post-flood behaviors showed that the data fit very well with the model: CFI = 0.991, TLI = 0.988, χ^2^/*df* = 1.27, and RMSEA = 0.026 (see Fig. 5). Furthermore, the saturation coefficients were all significant.

### Multiple correspondence analysis

**Fig. 6:**
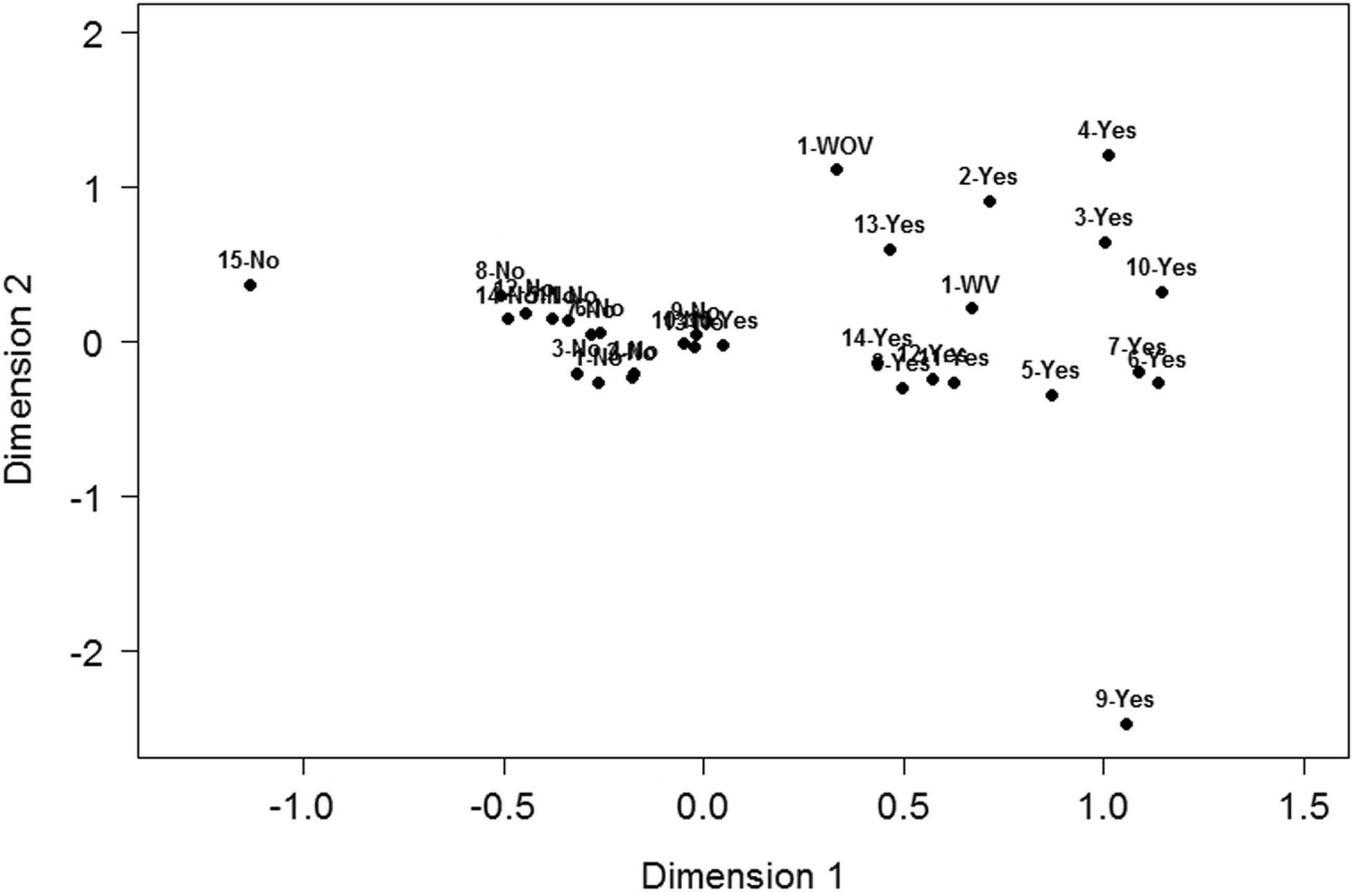
Projection of the active variables composing the index of pre-alert adaptation in the multiple correspondence analysis. Legend: 1-No: No list of belongings made, 1-SV: List of belongings made without video or photos, 2-AV: List of belongings made with video or photos. For all the other variables, “yes” represents the preventive modality and “no,” the non-preventive modality: 2-Make a plan for evacuating your neighborhood; 3-Know how to shut off the electricity or the water; 4-Inquire about how to better prepare for a flood or to make your home more flood-resistant; 5-Inquire about the consequences that a flood could have on your physical or mental health; 6-Waterproof the foundations; 7-Raise the baseboard heaters or electrical outlets on the walls; 8-Replace water-sensitive flooring with a waterproof material; 9-Install a backwater valve; 10-Relocate the home elsewhere on the property; 11-Make other changes to the home; 12-Change the landscape to help water runoff; 13-Check to be sure the foundation drain is not blocked; 14-Make other landscape changes; 15-Own a water pump

A multiple correspondence analysis was then performed on each index. For the preventive adaptation index, the results revealed that the total inertia explained by the first dimension was 98.68%, and the confirmatory factor analysis results showed that the index was unidimensional. Indeed, the projected coordinates of the active variables (i.e., the 15 behaviors) showed that all the response categories indicating that people adopt the behaviors (e.g., I usually or always adopt the behavior) were situated on the left side of the plot and all the responses indicating that people do not adopt the behaviors (e.g., I never adopt the behavior; I rarely adopt the behavior) were situated on the right side of the plot (see Fig. 6).

Multiple correspondence analyses were also performed on the four other indices. The results indicated that these indices were unidimensional, the first dimension obtained explaining a high percentage of the total inertia in each case: 77.43% (at the time of the alert), 69.50% (during a flood requiring evacuation), 94.30% (during a flood not requiring evacuation), and 91.86% (post-flood) of the total inertia. In Fig. 6, the affirmative answers (yes) indicate that the respondents who adopt the adaptive behavior are at the right of the axis, whereas the negative answers (no) are at the left of the axis. By examining the table presenting the contribution of the modalities (data not presented), one can determine which behavior is the most closely related to the index.

Finally, for each of these four indices, the projected coordinates of the behaviors measured showed that all the response categories indicating that people adopt the behaviors were situated on the left side of the plot and that the responses indicating that people do not adopt the behaviors were situated on the right side of the plot (data not shown; see online resource 5).

### Indices validity test

The purpose of this statistical analysis was to examine the relationship between the five behavioral indices of flood adaptation and theoretically related variables [18, 41]. As previously indicated, in this study, this indicator varied according to the subsample of the population under study. When the index to be created concerned only the population having experienced a flood (behaviors to adopt during a flood not requiring evacuation, behaviors to adopt during a flood requiring evacuation, and post-flood behaviors), the variable corresponded to the adverse health impacts felt during a flood. In the cases where the index targeted the entirety of the sample (preventive behaviors to adopt before the alert and behaviors to carry out after the alert is issued), the variable used was the perceived risk of being flooded in the next five years.

For each index, adaptation scores were generated using the coordinates of the behaviors obtained through the multiple correspondence analysis. These scores ranged from − 5 to + 5 and displayed a quasi-normal distribution (for more details on how these scores were generated, see Greenacres [38]) and were dichotomized as follows: individuals who adapted well to flooding (score < 0) and those who adapted less well (score > 0).

We tested the validity of each of the flood adaptation indices using a tetrachoric correlation with the measurement of self-reported health impacts or the perceived risk of being flooded in the coming years. Using a nominal-type polytomous logistic analysis [42], we also calculated the prevalence of health impacts (at-risk group, lower risk group) or risk perception (low perceived risk, high perceived risk) according to the adaptation level as measured by the dichotomized indices. The results (see Tables 7 and 8) showed that four out of the five indices had good validity.

**Table 7:** Prevalence of the perceived risk of being flooded in the next five years (indices 1 and 2)

Level of Adaptation to Flooding	% Who Reported a Perceived Risk of Being Flooded	Confidence Interval (95%)	Coeff. of Variation (%)	Odds Ratio	Confidence Interval (95%)	Pr > χ^2^
Index 1: Pre-alert behaviors Correlation with level of adaptation to flooding = 0.25, *p* < .05						
Adaptation	34.14	[30.38–37.91]	5.62	2.02	[1.63–2.51]	< 0.0001
Non-adaptation	20.39	[17.94–22.84]	6.12	1.00		
Index 2: Behaviors to carry out after the alert is issued Correlation with level of adaptation to flooding = 0.23, *p* < .05						
Adaptation	56.70	[50.41–62.98]	5.63	1.80	[1.30–2.51]	0.0004
Non-adaptation	42.05	[36.11–48.00]	7.18	1.00

**Table 8:** Prevalence of the reported adverse health impacts felt during or after a flood(indices 3, 4, and 5)

Level of Adaptation to Flooding	% Who Reported Adverse Health Impacts	Confidence Interval (95%)	Coeff. of Variation (%)	Odds Ratio	Confidence Interval (95%)	Pr > χ^2^
Index 3: Behaviors to adopt during a flood not requiring evacuation Correlation with level of adaptation to flooding = 0.22, *p* < .05						
Adaptation	24.83	[19.62–30.04]	10.66	1.85	[1.23–2.77]	0.0029
Non-adaptation	15.15	[10.60–19.71]	15.27	1.00		
Index 4:Behaviors to adopt during a flood requiring evacuation Correlation with level of adaptation to flooding = −0.18, n.s.						
Adaptation	32.27	[20.79–43.75]	17.86	0.62	[0.29–1.35]	0.2307
Non-adaptation	43.39	[27.80–58.97]	17.83	1.00		
Index 5: Post-flood behaviors Correlation with level of adaptation to flooding = 0.36, p < .05						
Adaptation	37.27	[29.83–44.72]	10.12	2.78	[1.72–4.50]	0.0001
Non-adaptation	17.61	[11.46–23.76]	17.68	1.00		

Indeed, the indices corresponding to pre-alert preventive behaviors, behaviors to carry out after the alert is issued, behaviors to adopt during a flood not requiring evacuation, behaviors to adopt during a flood requiring evacuation, and post-flood behaviors were significantly and positively correlated with their respective related variable. However, no significant correlation was found between the index measuring adaptation during a flood not requiring evacuation and the perceived risk of being flooded in the coming years.

The results of the odds ratio analyses were consistent with the correlation results. According to the indices, the prevalence of perceived risk of being flooded over the next five years was higher in the group that adapted than in the one that did not adapt, except in the case of the index measuring adaptation during a flood not requiring evacuation. For instance, the results suggested that 37.27% of the respondents adapted well to post-flooding (score < 0) versus 17.61% who adapted less well (score > 0): odds ratio = 1.37, *p* = 0.0011.

#### Reported health problems or diseases

The results showed that 115 of the 797 (14.45%) people having experienced at least one flood in their current home reported that their physical health had been moderately or greatly affected by the flood. Among them, 50 (43.51%) had consulted a health professional (e.g., doctor, physiotherapist, chiropractor) because of these physical health problems. The most frequently reported problems are presented in Table 9.

**Table 9:** Most frequently reported physical health problems

Bone disease or pain .....................................................................................................................12	
Illness, such as flu, virus, cough ................................................................................................8	
Back, leg or muscle pain .............................................................................................................18	
Headaches, dizziness...........................................................................................................................3	
Respiratory and heart problems (asthma) .........................................................................7	
Exhaustion, lack of sleep .............................................................................................................31	
Digestive problems ............................................................................................................................4	
Others ...........................................................................................................................................................8	
No answer ..............................................................................................................................................25	

The results showed also that 136 of the 797 (17.08%) people having experienced at least one flood in their current home reported that their mental health had been moderately or greatly affected negatively by the flood. Among them, 35 (25.42%) had consulted a health professional (e.g., doctor, psychologist) because of these mental health problems. The most frequently reported problems are presented in Table 10.

**Table 10:** Most frequently reported mental health problems

Anxiety, distress, PTSD ...................................................................................................................23	
Depression, bipolar disorder .....................................................................................................15	
Fear, concern, uncertainty, insecurity ................................................................................57	
Sleep disorder ......................................................................................................................................13	
Frustration, anger .................................................................................................................................4	
Sorrow, sadness, low morale ......................................................................................................3	
Discouragement, demotivation, depression, disgust.................................................8	
No answer ..............................................................................................................................................14	

#### Relationship between the five behavioral indices of flood adaptation

Our results revealed that the five behavioral indices were moderately and significantly correlated (see Table 11). The magnitude of the correlations varied between 0.23 and 0.47. Table 11 shows that index 3 (behaviors to adopt during a flood requiring evacuation) is the index least correlated with the other indices.

**Table 11:** Correlations between the five indices of flood adaptation

Indices	1	2	3	4	5
1. Pre-alert behaviors	–				
2. Behaviors to carry out after the alert is issued	.43	–			
3. Behaviors to adopt during a flood not requiring evacuation	.39	.34	–		
4. Behaviors to adopt during a flood requiring evacuation	.23	.20	None^a^	–	
5. Past-flood behaviors	.43	.30	.48	.27	–

^a^There is no correlation between indices 3 and 4 because respondents could not be in both flooding situations

#### Impact of socio-demographic variables

Regarding socio-economic data, no statistically significant differences were found between genders for the five adaptation indices. The results also showed that only age had an impact during a flood not requiring an evacuation (index 3), where people aged 70 to 79 had lower rates of adaptation (37.83%) than those aged 40 to 49 (65.99%). Individuals without a school diploma showed lower rates of adaptation (32.94%) during an alert (index 2) compared to respondents who had graduate degrees (57.32%). The same pattern was observed during a flood not requiring an evacuation (index 3): individuals without a school diploma showed lower rates of adaptation (40%) compared to respondents who had graduate degrees (57.42%). Finally, household income had a significant impact on preventive adaptation (index 1): people who made less than CAD$20,000 per year had lower adaptation rates (22.31%) than those earning between CAD$40,000 and CAD$60,000 (38.65%) and those earning more than CAD$80,000 (40%). Household income also had a significant impact on post-flood adaptation (index 5): people who made less than CAD$20,000 per year had lower adaptation rates (25.63%) than those earning more than CAD$80,000 (61.11%).

## Discussion

Given that floods are common in the province of Quebec (Canada) and that their impacts on overall health are well known, municipal and public health authorities have established protective measures to foster the adaptation of at-risk citizens. However, to our knowledge, there has been no surveillance of these flood adaptation behaviors to date. Therefore, it is impossible to determine whether the measures promoted by the authorities are actually being adopted by the population and whether they are effective for reducing the perceived health impacts. In a context of climate change where the risk of flooding is growing, it seemed important to bridge this gap.

This investigation is a substantive-methodological synergy with a focus on the construct validity (factor structure, reliability, correlation with a related variable) of five flood adaptation indices for people living in or bordering a flood zone. Across a series of psychometric analyses, the results showed that these flood adaptation indices were able to properly measure a vast range of adaptive behaviors, that is, behaviors to adopt according to the chronology of events: before the alert, at the time of the alert, during a flood requiring evacuation, during a flood not requiring evacuation, and after the flood. At a more practical level, a key contribution of this research relates to the development not only of valid indices, but also of parsimonious ones based on simple actions. These actions are recommended by public health agencies to protect individuals against flooding and should consequently reduce the severity of adverse effects on mental and physical health [22–29]. The results of this study show that a significant proportion of respondents with flood experience reported medium to high negative impacts of flooding on their physical and mental health. As nearly half of them required treatment from a health professional, improving adaptation rates could help improve flood victims’ health outcomes and reduce some of the strain put on the healthcare system. The need for increased flood education and better adaptation at the community level in Canada’s population has already been noted by Burton, Rabito et al. [43]. These authors also emphasized the importance of better assessments of current environmental disaster resilience strategies. Because the completion of these five flood adaptation questionnaires is not time-consuming, they could be used in community and national surveys to monitor individuals’ adaptation to flooding. They could also help better target the characteristics of the people who do not adapt as well and thus define the required interventions more clearly. In this regard, because our data shows that those in the lowest income bracket adopt fewer preventive and post-flood behaviors, some interventions may be required to remove the economic barriers preventing this group from attaining desirable adaptation rates. For example, subsidies could be offered for adapting one’s basement for potential floods. Over time, this kind of monitoring could enable public health agencies to better identify protective behaviors to incorporate in health promotion campaigns.

Another contribution of this study relates to the unidimensionality of the indices. The total inertia explained by the first dimension in the multiple correspondence analysis and the good fit indices obtained in the confirmatory factor analysis both support the unidimensionality of the indices. More precisely, these results support the idea that a single latent trait was able to account for most of the variance shared among the behaviors included in the indices. Consequently, items belonging together in each index appear to capture differences in the same underlying construct, namely flood adaptation. From a practical perspective, this unidimensionality is extremely important because the behaviors underlying a given index should reflect individual differences regarding this specific construct, even if it is related to another one (e.g., resilience). Otherwise, the interpretation of the index score as being representative for the construct mentioned in the hypothesis could be wrong [44]. Some researchers may argue that flood adaptation could be multidimensional. In fact, this is a critical issue to resolve theoretically because it has a major influence on the interpretation of the index scores. Consequently, we performed factor analyses to test multidimensional models (data not shown). We found that the multidimensional models were not as suitable (i.e., poorer statistical fit indices) as the unidimensional model.

The fact that four out of the five indices have good validity is also noteworthy. The validity of the indices was supported by their correlations with the perceived risk of being flooded in the next five years or self-reported adverse health impacts of flooding according to the adaptation indices. The results of this study showed that people who perceive a risk of flooding in their home in the next five years adopt a few more preventive behaviors and adaptation behaviors at the time of the alert than those who perceive little or no risk at all. This is consistent with the results of various studies on the subject [17]. The results also revealed that people who feel more adverse effects on their physical or mental health adopt more adaptive behaviors during a flood not requiring evacuation as well as post-flood adaptation behaviors than those who feel little or no adverse effects on their health. This finding is similar to the results obtained in other studies on adaptation to extreme climatic events [45, 46]. Conversely, the results showed that the index of adaptation at the time of evacuation does not present a good validity. However, this result must be interpreted with caution because of the small sample size. Indeed, only 124 of the 1951 respondents had experienced such an evacuation, which is not surprising given that evacuated people rarely represent the majority of those affected by a flood [47]. Regardless of this reservation, the values of the fit indices of the confirmatory factor analysis show that the model fits the data well. This leads us to believe that this index is nevertheless usable. Further studies are needed to validate this hypothesis.

Finally, the five behavioral indices of flood adaptation were found to be significantly related. This suggests that individuals who adapt well to flooding before the alert adapt well also at the time of the alert, during a flood not requiring an evacuation, during a flood requiring an evacuation, and after the flood. The results indicate, however, that this finding is less likely to be true for the behaviors to adopt during a flood requiring an evacuation. This finding highlights the fact that the authorities must create conditions facilitating performance of the behaviors to adopt after a flood and must remove any potential barriers to their adoption (e.g., forming environmental health teams whose members have learned to quickly mobilize to guide flood victims who are required to evacuate the neighborhood).

Despite its strengths, this study has some limitations. The first is its reliance on self-reports of flood adaptation and the possibility of participants having overestimated the extent to which they performed these socially desirable behaviors. Second, given the low response rate obtained, the sample cannot be considered representative of all individuals living in an at-risk area. For example, we cannot reject the hypothesis that the sample obtained was composed mainly of individuals who were more concerned about flooding. However, those who agreed to participate in the study responded to the great majority of the questions. Third, to ensure that these validated indices can be used in other regions or countries concerned with flooding conditions, the measurement invariance in those areas must first be demonstrated in independent samples. Measurement invariance tests would be used to evaluate the extent to which measurement properties of the adaptation indices generalize across various countries and cultures and over various periods. A lack of measurement invariance of the indices could lead to rather serious consequences. They could include inaccurate assessments of the evolution of adaptive behaviors during flooding over the years and of the real impact of a behavior change strategy (e.g., communication campaign) on people’s motivation to adopt flood adaptation behaviors. Additionally, there is always a risk that some behaviors composing each index become a habit. This could reduce the variance of these behavioral indicators and consequently their power to differentiate people who adapt from those who do not adapt as well. Thus, the validity of each index should be tested in the future to examine the relevance of the behaviors composing these indices. Finally, the validity analysis conducted did not involve any assumption of causality or directionality. Future research should verify the link of causality between self-reported adverse health effects and perceived risk of being flooded in the future and adaptive behaviors.

## Conclusion

In conclusion, these findings demonstrate that the five flood adaptation indices for people living in or bordering a flood zone have a sound factor structure, good reliability, and construct validity. Although additional tests of the measurement invariance of the indices across various countries are needed, this study underscores their validity. Therefore, researchers, public health agencies, and professionals can use them to monitor the evolution of individuals’ adaptive behaviors during floods.

## Additional files

Additional file 1: **Online resource 1.** Discrimination indices for each behavior at the time of the alert. Results of the item analysis for the index of adaptation at the time of the alert. (DOCX 15 kb)

Additional file 2: **Online resource 2.** Discrimination indices for each behavior during a flood not requiring an evacuation. Results of the item analysis for the four behaviors from the index of adaptation at the time of the flood not requiring an evacuation. (DOCX 14 kb)

Additional file 3: **Online resource 5.** Projection of the active variables in the multiple correspondence analysis for each index of adaptation. Item characteristic curves from the non-parametric item analysis models for each of the non-preventive index of adaptation. (DOCX 164 kb)

Additional file 4: **Online resource 3.** Discrimination indices for post-flood behaviors. Results from the item analysis for the 10 behaviors from the post-flood adaptation index. (DOCX 14 kb)

Additional file 5: **Online resource 4.** Behaviors removed from the index because their correlation with another behavior was too high. Table of the eight behaviors removed from the pre-alert preventive index because their correlation with another behavior was too high. (DOCX 15 kb)

## Abbreviations

CFI: comparative fit index
PTSD: post-traumatic stress disorder
RMSEA: root mean square error of approximation
TLI: Tucker-Lewis index
