# Supplementary material for: Development and validation of five behavioral indices of flood adaptation
Source: BMC Public Health. 2019 Feb 28;19:245. doi: 10.1186/s12889-019-6564-0 (PMC6394037; doi:10.1186/s12889-019-6564-0)
Supplement: Supplementary file 1 — Online resource 1. Discrimination indices for each behavior at the time of the alert. Results of the item analysis for the index of adaptation at the time of the alert. (DOCX 15 kb) [file 12889_2019_6564_MOESM1_ESM.docx]

Online resource 1. Discrimination indices for each behavior at the time of the alert

| Adaptive behaviors | Discrimination index | 99% CI |
| --- | --- | --- |
| 1. Move your lawn or patio furniture or your vehicle to higher ground | 0.90 | [0.63-1.16] |
| 1. Store items or furniture higher or on a higher floor | 1.12 | [0.83-1.41] |
| 1. Block the basement drain | 0.52 | [0.21-0.83] |
| 1. Cut off the electricity if requested by the authorities | 0.77 | [0.48-1.07] |
| 1. Waterproof the doors and windows with plastic tape | 1.47 | [0.96-1.97] |
| 1. Block the outside air inlets like the one for the clothes dryer, the range hood, the air exchanger, etc. | 1.64 | [1.00-2.27] |
| 1. Put sandbags on the property | 1.67 | [1.27-2.08] |
| 1. Other measures to prevent water from entering the home | 0.66 | [0.02-1.31] |
| 1. Check regularly if the risk of flooding has increased or decreased | 0.62 | [0.38-0.86] |
| 1. Help your neighbors implement their protective measures like putting sandbags | 1.56 | [1.17-1.94] |
