# Supplementary material for: Development and validation of five behavioral indices of flood adaptation
Source: BMC Public Health. 2019 Feb 28;19:245. doi: 10.1186/s12889-019-6564-0 (PMC6394037; doi:10.1186/s12889-019-6564-0)
Supplement: Supplementary file 2 — Online resource 2. Discrimination indices for each behavior during a flood not requiring an evacuation. Results of the item analysis for the four behaviors from the index of adaptation at the time of the flood not requiring an evacuation. (DOCX 14 kb) [file 12889_2019_6564_MOESM2_ESM.docx]

Online resource 2. Discrimination indices for each behavior during a flood not requiring an evacuation

| Adaptive behaviors | Discrimination index | 99% CI |
| --- | --- | --- |
| 1. Boil the water or use bottled water | 0.745 | [0.493-0.998] |
| 1. Wear rubber gloves to handle items in contact with the flood water | 2.299 | [1.766-2.833] |
| 1. Wear rubber boots to walk in the flood water | 1.270 | [0.936-1.605] |
| 1. Install a pump to evacuate water from your home | 0.815 | [0.555-1.075] |
