# Supplementary material for: Development and validation of five behavioral indices of flood adaptation
Source: BMC Public Health. 2019 Feb 28;19:245. doi: 10.1186/s12889-019-6564-0 (PMC6394037; doi:10.1186/s12889-019-6564-0)
Supplement: Supplementary file 3 — Online resource 5. Projection of the active variables in the multiple correspondence analysis for each index of adaptation. Item characteristic curves from the non-parametric item analysis models for each of the non-preventive index of adaptation. (DOCX 164 kb) [file 12889_2019_6564_MOESM3_ESM.docx]

Online resource 3. Discrimination indices for post-flood behaviors

| Adaptive behaviors | Discrimination index | 99% CI |
| --- | --- | --- |
|  |  |  |
| 1. Have the condition of the electrical installation and heating appliances checked | 2.134 | [1.582-2.686] |
| 1. Replace the refrigerator insulation if it is wet or replace the appliance | 0.996 | [0.675-1.318] |
| 1. Disinfect the contaminated rooms | 2.079 | [1.463-2.695] |
| 1. Sterilize all kitchen items contaminated by the flood water | 1.673 | [1.209-2.138] |
| 1. Discard items in contact with the flood water | 1.934 | [1.425-2.443] |
| 1. Wear rubber gloves to handle items in contact with the flood water | 1.309 | [0.925-1.694] |
| 1. Check if mold has developed | 0.314 | [-0.039-0.667] |
| 1. Make a list of the damages caused to the home | 1.804 | [1.381-2.227] |
| 1. Update your emergency kit | 0.384 | [0.091-0.677] |
| 1. Attend citizens’ meetings concerning the flood | 1.264 | [0.882-1.645] |
