# Supplementary material for: Development and validation of five behavioral indices of flood adaptation
Source: BMC Public Health. 2019 Feb 28;19:245. doi: 10.1186/s12889-019-6564-0 (PMC6394037; doi:10.1186/s12889-019-6564-0)
Supplement: Supplementary file 4 — Online resource 3. Discrimination indices for post-flood behaviors. Results from the item analysis for the 10 behaviors from the post-flood adaptation index. (DOCX 14 kb) [file 12889_2019_6564_MOESM4_ESM.docx]

Online resource 4. Behaviors removed from the index because their correlation with another behavior was too high

| Behaviors removed | Number of behaviors in the index | Reasons | Model fit | | | |
| --- | --- | --- | --- | --- | --- | --- |
|  |  |  | CFI | TLI | χ^2^/*dl* | RMSEA |
| None – Base model | 23 |  | 0.794 | 0.773 | 7.88 | 0.059 |
| Know how to shut off the water | 22 | Too highly related to “know how to cut off the electricity” (r = 0.663) | 0.818 | 0.799 | 7.49 | 0.058 |
| Raise the foundations | 21 | Too highly related to “raise the door sills” (r = 0.743) | 0.834 | 0.816 | 6.66 | 0.054 |
| Make a plan for evacuating your home | 20 | Too highly related to “make a plan for evacuating your neighborhood” (r = 0.554) | 0.861 | 0.844 | 5.91 | 0.050 |
| Do drainage work around the home | 19 | Too highly related to “check to be sure the foundation drain is not blocked” (r = 0.611) | 0.883 | 0.868 | 4.87 | 0.045 |
| Raise the door sills | 18 | Too highly related to “waterproof the foundations (r = 0.614) | 0.895 | 0.881 | 4.36 | 0.041 |
| Reduce the area of surfaces that are not waterproof | 17 | Highly related to “change the landscape to help water runoff” (r = 0.485) | 0.897 | 0.883 | 4.54 | 0.043 |
| Have an emergency kit | 16 | Highly related to “have a list of emergency telephone numbers” (r = 0.446) and to “make a plan for evacuating your neighborhood (r = 0.484) | 0.906 | 0.892 | 4.13 | 0.040 |
| Have a list of emergency phone numbers | 15 | Highly related to “make a list of your belongings” (r = 0.441) and to “make a plan for evacuating your neighborhood” (r = 0.450) | 0.924 | 0.911 | 3.50 | 0.036 |
