# Supplementary material for: Development and validation of five behavioral indices of flood adaptation
Source: BMC Public Health. 2019 Feb 28;19:245. doi: 10.1186/s12889-019-6564-0 (PMC6394037; doi:10.1186/s12889-019-6564-0)
Supplement: Supplementary file 5 — Online resource 4. Behaviors removed from the index because their correlation with another behavior was too high. Table of the eight behaviors removed from the pre-alert preventive index because their correlation with another behavior was too high. (DOCX 15 kb) [file 12889_2019_6564_MOESM5_ESM.docx]

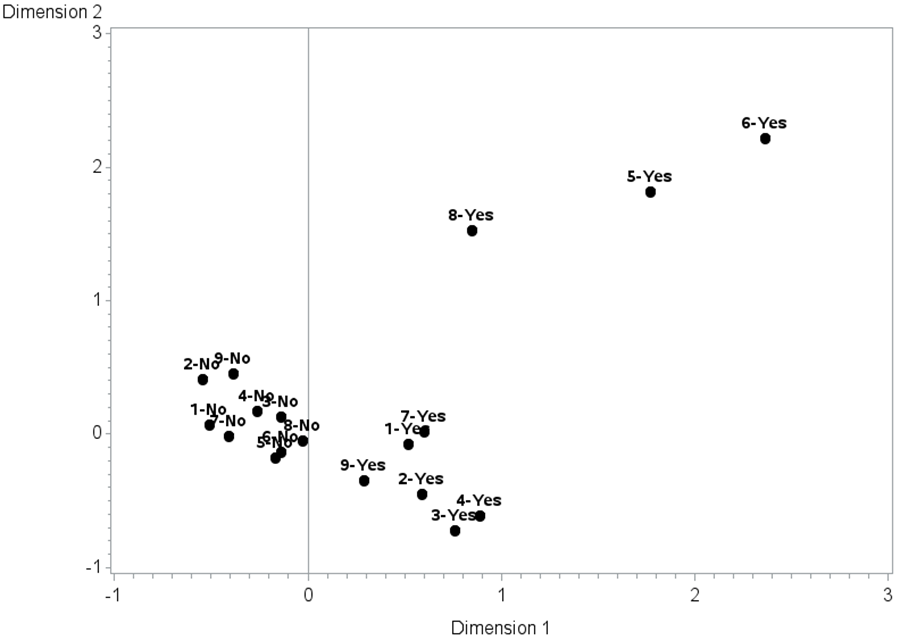


Legend: For all the variables, “yes” represents the adaptative modality and “no,” the non-adaptive modality:
1-Move your lawn or patio furniture or your vehicle to higher ground; 2- Store items or furniture higher or on a higher floor; 3-Block the basement drain; 4-Cut off the electricity if requested by the authorities; 5-Waterproof the doors and windows with plastic tape; 6-Block the outside air inlets like the one for the clothes dryer, the range hood, the air exchanger, etc.; 7-Put sandbags on the property or help your neighbors implement their protective measures; 8-Other measures to prevent water from entering the home; 9-Check regularly if the risk of flooding has increased or decreased.

Online resource 5. Projection of the active variables in the multiple correspondence analysis for the index of adaptation at the time of the alert


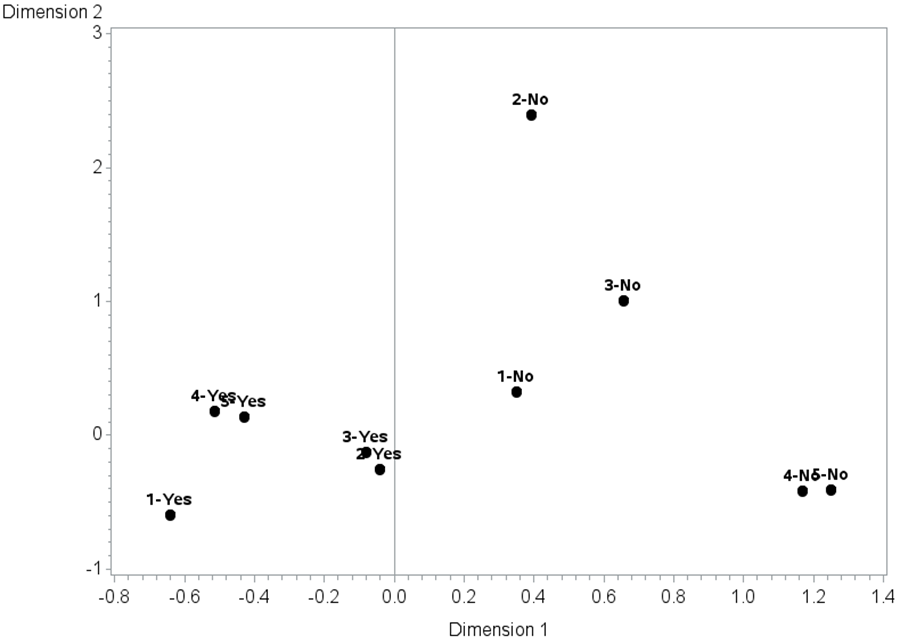


Legend: For all the variables, “yes” represents the adaptative modality and “no,” the non-adaptative modality: 1-Bring your emergency kit, including your medication; 2-Lock the doors when leaving; 3-Tell your loved ones where you can be easily reached; 4-Use the route indicated by the authorities to evacuate the neighborhood; 5-Wait for the authorities’ permission before returning home.

Online resource 5. Projection of the active variables in the multiple correspondence analysis for the index of adaptation during the evacuation


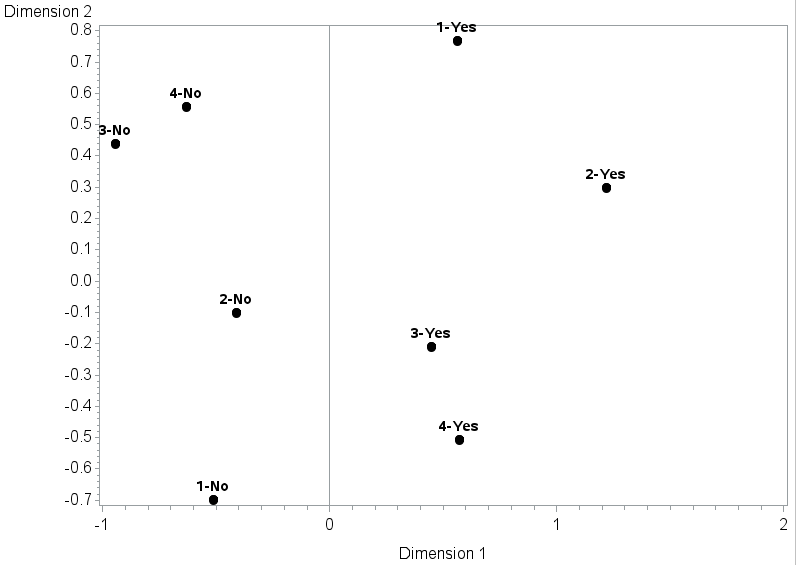


Legend: For all the variables, “yes” represents the adaptive modality and “no,” the non-adaptative modality: 1-Boil the water or use bottled water; 2-Wear rubber gloves to handle items in contact with the flood water; 3-Wear rubber boots to walk in the flood water; 4-Install a pump to evacuate the water from your home.

Online resource 5. Projection of the active variables in the multiple correspondence analysis for the index of adaptation during a flood not requiring evacuation


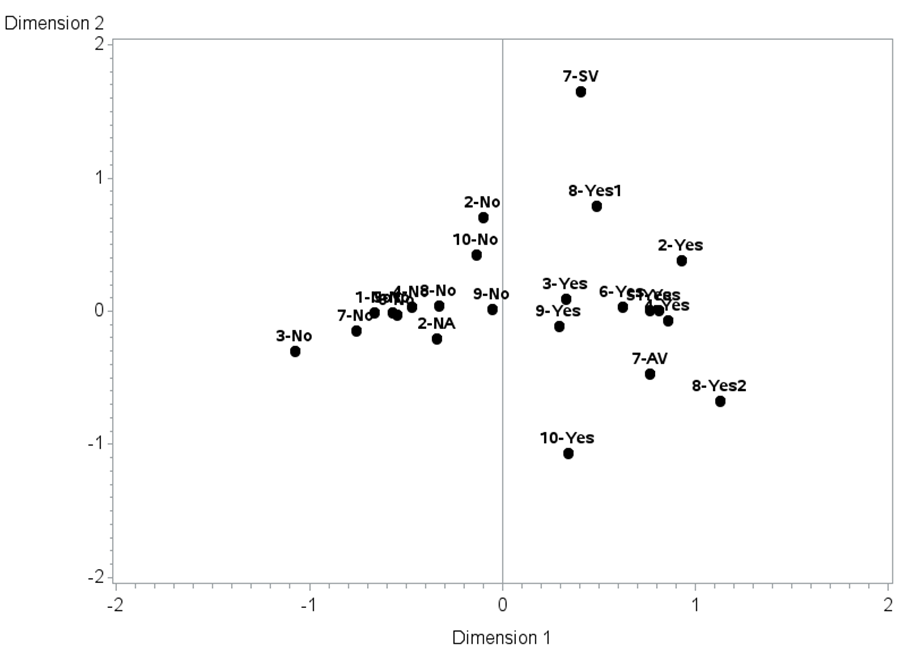


Legend: 1-Have the condition of the electrical installation and heating appliances checked (yes, no); 2-Replace the refrigerator insulation if it is wet or replace the appliance (yes, no, not applicable); 3-Disinfect the contaminated rooms (yes, no); 4-Sterilize all kitchen items contaminated by the flood water (yes, no); 5-Discard all items that were in contact with the flood water (yes, no); 6-Wear rubber gloves to handle items in contact with the flood water (yes, no); 7-Make a list of the damages caused to the home (AV: with video or photos, SV: without video or photos, no); 8-Attend citizens’ meetings concerning the flood (yes2: 2 meetings or more, yes1: 1 meeting; no: no meetings); 9-Check if mold has developed (yes, no); 10-Update your emergency kit (yes, no).

Online resource 5. Projection of the active variables in the multiple correspondence analysis for the index of post-flood adaptation
